# Profiling of rare immune cell populations and integrative analysis identify immune ecotypes in newly diagnosed meningiomas

**DOI:** 10.1186/s40478-026-02276-0

**Published:** 2026-03-18

**Authors:** Catharina Lotsch, Nicoletta Giuliani Canizales, Lena Jassowicz, Carmen Rommel, Mandy Barthel, Katrin Lamszus, Almuth F. Kessler, Niels Grabe, Mario Loehr, Ralf Ketter, Christian Senft, Sybren L. N. Maas, Philipp Sievers, Manfred Westphal, Matthias Simon, Andreas von Deimling, Andreas Unterberg, Sandro M. Krieg, Felix Sahm, Rolf Warta, Christel Herold-Mende

**Affiliations:** 1 Division of Experimental Neurosurgery, Department of Neurosurgery, Medical Faculty Heidelberg, Heidelberg University, INF400, 69120 Heidelberg, Germany; 2 Department of Otorhinolaryngology, Head and Neck Surgery, Medical Faculty Heidelberg, Heidelberg University, Heidelberg, Germany; 3 Institute for Tumor Biology, University Medical Center Hamburg-Eppendorf, Hamburg, Germany; 4 Department of Neurosurgery, University Hospital Würzburg, Würzburg, Germany; 5 Hamamatsu Tissue Imaging and Analysis Center (TIGA), BIOQUANT, Heidelberg University, Heidelberg, Germany; 6 Department of Neurosurgery, Medical School, Saarland University, Homburg, Germany; 7 Department of Neurosurgery, Jena University Hospital, Friedrich-Schiller-University, Jena, Germany; 8 Department of Pathology, Leiden University Medical Center, Leiden, The Netherlands; 9 Department of Pathology, Brain Tumor Center, Erasmus MC Cancer Institute, University Medical Center Rotterdam, Rotterdam, The Netherlands; 10 Clinical Cooperation Unit Neuropathology, German Cancer Research Center (DKFZ), Heidelberg, Germany; 11 Department of Neuropathology, Institute of Pathology, Heidelberg University, Heidelberg, Germany; 12 German Cancer Consortium (DKTK), Heidelberg, Germany; 13 Department of Neurosurgery, University Hospital Bonn, Bonn, Germany; 14 Department of Neurosurgery, Ev. Klinikum Bethel, University of Bielefeld Medical Center OWL, Bielefeld, Germany

**Keywords:** Meningioma, Immunobiology, DNA methylation, Prognosis, Immune ecotypes

## Abstract

Meningiomas (MGMs) are the most common primary intracranial tumors in adults with a substantial subset exhibiting aggressive clinical behavior. Immunotherapy represents a potential alternative treatment option, even though MGMs have traditionally been considered “immunologically cold” tumors. This study explored less characterized immune cell subsets —B cells, natural killer (NK) cells, and granulocytes— and their associations with major immune cell populations as well as their prognostic implications. For this purpose, we performed tissue cytometry analysis in a clinically well-annotated multi-center cohort of 97 newly diagnosed MGMs encompassing all WHO grades (1, 2, 3) and DNA methylation classes (benign, intermediate, malignant). Resulting infiltration data were integrated with previously published data on tumor-associated macrophages (TAMs) and tumor-infiltrating T lymphocytes (TILs) to identify MGM immune ecotypes. Overall, infiltration rates of B cells, NK cells, neutrophils, and eosinophils showed lower frequencies and varied widely across tumors. Notably, we observed significantly lower numbers of B cells in MGM with losses in chromosomal arms 10q and 22q, while lower number of T cells were found in patients with a loss of chromosomal arm 1p. In addition, NK cells and eosinophils were enriched in grade 1 and benign tumors, whereas neutrophils predominated in malignant cases. Despite their relatively low abundance, elevated neutrophil frequencies turned out to be an independent of prognostic factor for poor survival. Importantly, subsequent integration of TAM and TIL data derived from the same patient cohort unraveled five distinct immune ecotypes, each displaying characteristic immune cell infiltration patterns and differential survival outcomes. Altogether, this study provides an expanded overview of various rare immune cell subtypes in MGM and demonstrates their integration into different prognostic immune ecotypes, enabling better stratification in future clinical studies.

**Supplementary Information:**

The online version contains supplementary material available at 10.1186/s40478-026-02276-0.

## Introduction

Meningiomas (MGMs) represent the most prevalent neoplasms of the central nervous system (CNS) in adults, accounting for 41.7% of all CNS tumors in the United States, with incidence continuing to rise [32, 41]. Around 80% of these tumors are histologically considered benign and classified as grade 1 tumors by World Health Organization (WHO) 2021 grading, while approximately 18% are classified as CNS WHO grade 2 MGMs and 1–2% as grade 3 tumors [37, 41]. Even though MGMs have long been regarded as primarily benign brain tumors, a considerable proportion displays aggressive clinical behavior, resulting in 5-year recurrence rates of 10–15% for WHO grade 1 tumors, 50% for WHO grade 2 MGMs, and 90% for WHO grade 3 tumors [6, 26]. For many years, research on MGMs has primarily focused on the molecular pathogenesis of these tumors, leading to several molecular classifiers, mainly based on DNA methylation profiling, chromosome copy-number variations and genetic risk factors. These MGM classifiers have originally started with the Heidelberg classifier [35], and have substantially improved tumor diagnostics and patient stratification altogether [25, 40]. However, despite extensive research efforts, standard treatment modalities for MGM patients are still limited to surgical resection and radiotherapy. Systemic medical therapies remain largely experimental and have demonstrated only limited efficacy in treating refractory tumors. This also applies to clinical trials on T cell-based immune checkpoint blockade (ICB) therapy, which have not yet achieved any significant breakthrough in patients with recurrent MGMs [41, 43]. Nevertheless, these results were not surprising given that MGMs are characterized by a low tumor mutational burden and lower T cell infiltration, which are two major prerequisites for successful ICB treatment with a clinically meaningful outcome [42, 43]. Despite being viewed as an “immunologically cold” tumor compared to other malignancies, recent studies from our group have shown prognostic roles for CD3+ tumor-infiltrating T lymphocytes (TILs) and CD68 + tumor-associated macrophages (TAMs) in MGM. Particularly, high numbers of cytotoxic TILs and low numbers of immunosuppressive so-called M2-like TAMs were associated with improved progression-free survival (PFS) in patients with newly diagnosed MGMs [21, 34]. Moreover, using a DNA methylation-based deconvolution approach, the contrasting prognostic significance of TAMs and TILs was validated in an independent cohort: high TAM and low TIL infiltration present independent prognostic factors associated with poorer survival outcomes in MGM [21]. These results further emphasize the need to distinguish between favorable and unfavorable MGM immune-enriched subtypes and demand for a more in-depth profiling of the immunological landscape in MGMs in association with their methylation classes.

Previous studies analyzing the MGM immune compartment have primarily focused on the most prevalent immune cell subtypes, which are TAMs, microglia, T cells, and myeloid-derived suppressor cells (MDSCs) [20, 21, 31, 33, 34, 44, 46]. In contrast, data on the infiltration of B lymphocytes, natural killer (NK) cells, neutrophils and eosinophils in MGM are scarce [8, 10, 12, 45], particularly regarding their biological impact on disease progression and patient outcome. Therefore, in this study, we analyzed infiltration rates of B cells, NK cells, neutrophils and eosinophils in a large and clinically well-annotated study cohort of 97 newly diagnosed MGMs according to clinicopathological characteristics. Additionally, we integrated our previously published TAM and TIL data sets from the same patient cohort to unravel MGM immune ecotypes. Altogether our data revealed heterogeneous immune infiltration rates of neutrophils, eosinophils, B and NK cells across MGM specimens and further suggest relevant prognostic roles even for rarer immune cell subsets, such as neutrophils. Moreover, our integrative analysis discovered five distinct MGM immune ecotypes, each displaying characteristic immune cell infiltration patterns and differential survival outcomes, which might further influence immunotherapeutic treatment decisions in the future.

## Material and methods

### Sex as biological variable

In our study cohort, we included both male and female patients. Patient sex was considered as a biological variable and included as a clinically relevant prognostic cofactor in univariate and multivariate survival analyses.

### Tissue specimen collection

The study cohort consisted of *n* = 97 newly diagnosed MGMs (*n* = 29 WHO grade 1; *n* = 49 WHO grade 2; *n* = 19 WHO grade 3) that were obtained from male and female patients who underwent surgical resection in the Departments of Neurosurgery at University Hospitals Heidelberg, Bonn, Hamburg, Homburg, Frankfurt, and Wurzburg (all Germany) as part of the “FORAMEN” and “KAM” consortia [21, 34]. Patients were included between 2003 and 2015. Written informed consent was obtained from all patients. After surgical removal, tumor specimens were immediately snap-frozen and stored at − 80 °C until further processing and cryosectioning. For subsequent analyses, tumor cell content ≥ 60% was confirmed for all MGM specimens by an experienced neuropathologist (AvD) using hematoxylin & eosin-stained slides whereas tumor samples with a tumor cell content < 60% and/or with high necrosis were excluded. Clinical data were collected using a detailed questionnaire by an experienced study nurse (MB) and are summarized in Table 1.

**Table 1:** Clinicopathological characteristics of newly diagnosed meningiomas

	Whole cohort (*n* = 97)		
Variable	*n*	Patients (%)	Median (range)
*Sex*			
Male	35	36.1	
Female	62	63.9	
Age at 1st diagnosis			61.0 (21.0–87.6)
*WHO grade*			
WHO grade 1	29	29.9	
WHO grade 2	49	50.5	
WHO grade 3	19	19.6	
*Subtype*			
Transitional	10	10.3	
Fibrous	8	8.2	
Meningothelial	7	7.2	
Secretory	1	1.0	
Atypical	41	42.3	
Anaplastic	9	9.3	
Rhabdoid	1	1.0	
Papillary	2	2.1	
NA	18	18.6	
*Localization*			
Convexity	36	37.1	
Falxial	12	12.4	
Skull base	22	22.7	
Parasagittal	13	13.4	
Tentorial	2	2.1	
Other	10	10.3	
NA	2	2.1	
*Resection grade*			
Simpson 1	52	53.6	
Simpson 2	31	32.0	
Simpson 3	12	12.4	
NA	2	2.1	
*Methylation class*			
Benign-1	13	13.4	
Benign-2	19	19.6	
Benign-3	3	3.1	
Intermediate-A	34	35.1	
Intermediate-B	12	12.4	
Malignant	16	16.5	
*Prospective recurrency*			
Non-recurring	48	49.5	
Prospectively recurring	39	40.2	
NA	10	10.3	
*Postoperative radiotherapy*			
Yes	25	25.8	
No	68	70.1	
NA	4	4.1	
*Postoperative chemotherapy*			
Yes	1	1.0	
No	94	96.9	
NA	2	2.1	
*PFS (months)*			70.9 (0.0–213.6)
WHO grade 1			96.9 (0.2–171.7)
WHO grade 2			64.5 (1.0–213.6)
WHO grade 3			27.3 (0.0–147.5)
*OS (months)*			103.6 (0.7–213.6)
WHO grade 1			143.9 (47.0–193.9)
WHO grade 2			86.2 (26.2–213.6)
WHO grade 3			29.6 (0.7–173.1)
*Follow-up time (months)*			86.6 (0.0–213.6)
WHO grade 1			117.7 (0.2–182.2)
WHO grade 2			85.8 (3.0–213.6)
WHO grade 3			27.0 (0.0–147.5)

n, number; NA, not available; MGM, meningioma; PFS, progression-free survival; OS, overall survival

### Multicolor immunofluorescence staining

Multicolor immunofluorescence stainings were performed on acetone-fixed cryosections (5-7 µm) of MGM specimens of the study cohort, as described previously [21, 34]. To quantify B cells, NK cells, neutrophils and eosinophils, we used a combination of primary antibodies specific for CD20 (mouse, Thermo Fisher Scientific Cat# 14-0202-82, RRID: AB_10734340), NKp46 (goat, R&D Systems Cat# AF1850, RRID: AB_355019), CD66b (mouse, BioLegend Cat# 305102, RRID: AB_314494), and eosinophil peroxidase (EPX; mouse, Abcam Cat# ab199005, clone EPO104). Primary antibodies were diluted with Antibody Diluent (Dako) and incubated for 1 h. As secondary antibodies, goat anti-mouse IgG AlexaFluor488 (Thermo Fisher Scientific), donkey anti-goat IgG AlexaFluor555 (Thermo Fisher Scientific), and goat anti-mouse IgM AlexaFluor647 (Thermo Fisher Scientific), were applied, respectively. Secondary antibodies were diluted with DPBS-containing DAPI (Thermo Fisher Scientific) at 1:1,000 to stain nuclei and incubated for 1 h. Human tonsil and nasal mucosal tissue were used for positive control stainings while isotype-matched antibodies served as negative controls.

### Tissue cytometry‑based image analysis

Tissue cytometry-based image analysis was performed in a semi-automated setup at the single-cell level, followed by phenotypic hierarchical clustering as previously described [21, 34]. Briefly, high-resolution, automated multi-image alignments (MIAs) of whole-tissue sections were acquired using an Olympus IX51 microscope equipped with an XM10 camera (Olympus) using a 20 × objective. Image acquisition was conducted using the Olympus cellSens Dimension Software (version 1.9). For analysis, automated detection and context-based quantification of immune cell infiltration by immunofluorescence markers were carried out using StrataQuest Software (version 5.0.1, TissueGnostics GmbH). Regions of interest (ROIs) were manually delineated based on histopathological features and section quality to exclude necrotic areas and tissue borders. ROI definition was performed in the slide overview utilizing software-integrated markup tools. Quantification was restricted to regions exhibiting a high tumor cell content (≥ 60%) while necrotic areas were excluded. Detected cells were visualized in scattergrams and classified according to predefined gating schemes based on nuclear and cell surface marker expression (Suppl. Fig. S1). The thresholds for positive and negative gating were validated through backward gating procedures. To ensure robust and reproducible quantification, strict analytical parameters were applied for nuclear size, staining intensity, and background thresholding. Cell nuclei were identified via DAPI staining and served as the origin for generating expansion masks encompassing the cytoplasm and extending to the cell membrane or cell surroundings. Lymphoid cells were detected as single-nucleus cells whereas nuclei detection was modified for multinucleated granulocytes to detect large multi-segmented nuclei. Based on these masks, immune cells were analyzed for cell surface marker expression, including NKp46 for NK cells, CD20 for B cells (Suppl. Fig. S1a), and CD66b for neutrophils and EPX for eosinophils (Suppl. Fig. S1b). For lymphoid cell detection, double positive cells for NKp46 and CD20 cells were subsequently excluded. For eosinophil detection, we first excluded the nuclear detection to correct for extracellular staining of EPX in the cell surroundings (Suppl. Fig. S1b). Neutrophils were defined as CD66b-positive and EPX-negative, whereas eosinophils were characterized as CD66b- and EPX-double-positive. For statistical analyses, cell counts were expressed as the percentage of total cell count (%TCC), defined as the total number of DAPI-positive nuclei without further cell type discrimination.

### Consensus clustering to determine immune ecotypes

We performed unbiased consensus clustering of tissue cytometry data visualized as a heatmap using R (version 4.3.2) and the ConsensusClusterPlus (version 1.66.0) and ComplexHeatmap (version 2.18.0) packages. Samples were used as items and log₁₀-transformed cell densities were used as features. Due to the potential loss of accuracy when quantifying very low cell numbers, densities of less than one cell per mm^2^ were considered equivalent (log₁₀ density ≤ 0). The following parameters were used for consensus clustering: The maximum number of clusters (maxK) was set to 8, the number of resampling iterations (reps) to 10,000, the item resampling proportion (pItem) to 0.8, the feature resampling proportion (pFeature) to 1.0, the clustering algorithm (clusterAlg) to k-means, and the distance metric (distance) to Euclidean. The optimal number of clusters was selected by examining the cumulative distribution function (CDF) of the consensus matrix and the relative change in area under the CDF curve (delta area).

### Copy number variation analysis

Copy number variations (CNVs) were inferred from Illumina 450 k and EPIC DNA methylation array IDAT files using *conumee2* (v2.1.2) in R. Raw data were processed with the SeSAMe pipeline (QCDPB protocol), and total probe intensities (methylated + unmethylated signals) were used for CNV calling. An initial run using package-internal controls (minfiData/minfiDataEPIC) was performed to estimate CNV burden, and samples with the lowest burden (|log₂ ratio|≥ 0.20) were selected as platform-specific pseudo-controls. These were used as reference in a second analysis run, excluding them from the query cohort. Following reference fitting, binning, and circular binary segmentation, coverage-weighted mean log₂ ratios were calculated for chromosomal arms based on hg19 cytoband annotations. The following arms were analyzed: 1p, 1q, 3p, 6q, 10q, 14q, 18q, and 22q. Arm-level losses were defined as mean log₂ ratio ≤ − 0.20 and gains as ≥ + 0.20. CNV calls were integrated with immune cell infiltration data.

### Statistical analysis

Data were analyzed by GraphPad (Version 9.0.0) or R (Version 4.4.1, survival package). Non-parametric data are presented with median values and differences between two groups were calculated using Mann–Whitney-U tests. Survival differences were visualized by Kaplan–Meier plots and compared by log-rank test. PFS analyses were only performed on patients who underwent gross total resection (GTR) and had a follow-up time of at least 60 months. Multivariate analysis was performed using Cox proportional hazard model. Normalized data (log10-transformed) are presented with mean values and multiple comparison tests were performed using ordinary one-way ANOVA tests. Comparison between contingency table groups (distribution of clinical features by immune ecotype) were performed by Fisher’s exact test. *P* values < 0.05 were considered statistically significant: *, *P* < 0.05; **, *P* < 0.01, ***, *P* < 0.001, ****, *P* < 0.0001.

## Results

### Rarer immune cell populations show heterogenous infiltration patterns in meningioma

**Fig. 1:**
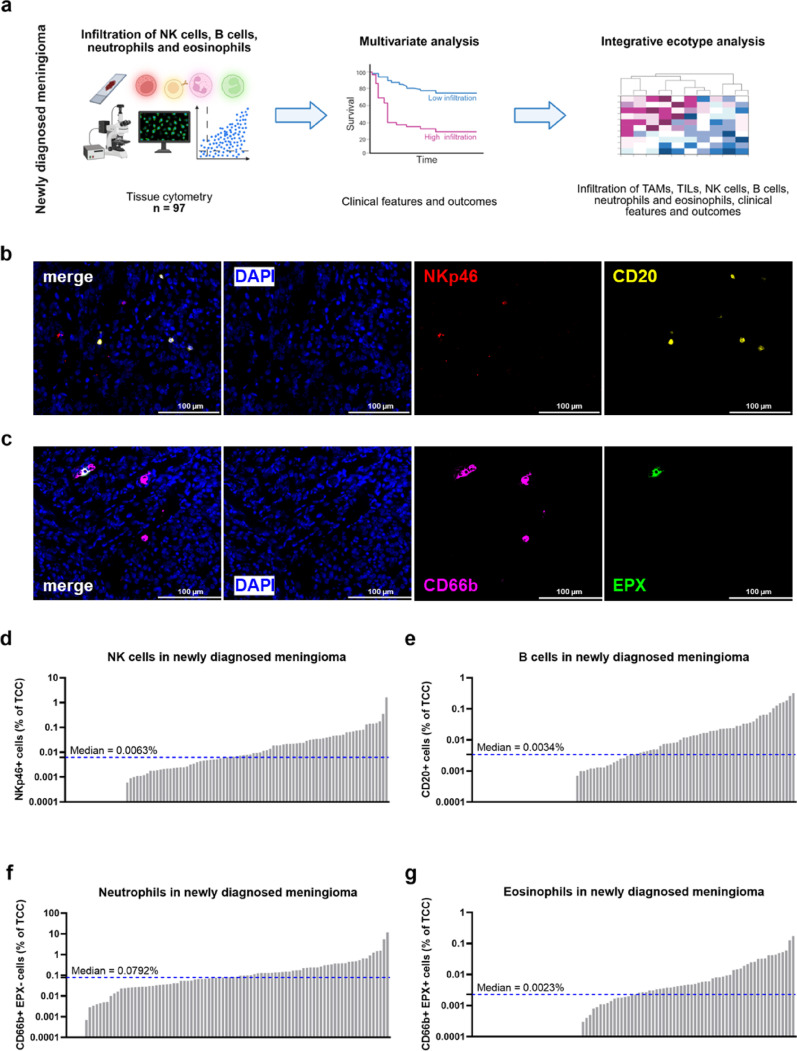
Analysis of rarer immune cell populations in newly diagnosed meningioma. **a** Schematic overview of the study workflow. Infiltration of NK cells, B cells, neutrophils and eosinophils was quantified by tissue cytometry in a study cohort of *n* = 97 newly diagnosed MGM specimens and analyzed in association to clinical parameters and survival outcomes. Data were finally integrated with previously published TAM and TIL infiltration data to analyze MGM immune ecotypes by consensus clustering. **b** Representative fluorescent images for NK and B cell staining. Nuclei stained with DAPI (blue), NK cells with NKp46 (red), and B cells with CD20 (yellow). **c** Representative fluorescent images for neutrophil and eosinophil staining. Nuclei stained with DAPI (blue), neutrophils with CD66b (pink), and eosinophils with EPX (green). **d** NK cell infiltration in newly diagnosed MGMs. **e** B cell infiltration in newly diagnosed MGMs. **f** Neutrophil infiltration in newly diagnosed MGMs. **g** Eosinophil infiltration in newly diagnosed MGMs. MGM, meningioma; NK, natural killer; TAM, tumor-associated macrophage; TCC, total cell count; TIL, tumor-infiltrating T lymphocyte

In this study, we investigated the frequencies of rarer immune cell populations in MGMs, such as B cells, NK cells, neutrophils and eosinophils, and their association with other more prevalent immune cell types, like TAMs and TILs, as well as their prognostic value (Fig. 1a). To this end, we performed tissue cytometry analyses in a cohort of *n* = 97 clinically well-annotated cases of newly diagnosed MGMs, which consisted of 29 WHO grade 1 MGMs, 49 WHO grade 2 tumors, and 17 WHO grade 3 cases (Table 1) and thus comprised a high frequency of clinically aggressive MGMs. MGM specimens were additionally classified by DNA methylation profiling using the Heidelberg classifier [35]: the study cohort comprised 35 benign (ben) tumors (*n* = 13 ben-1, *n* = 19 ben-2, *n* = 3 ben-3), 46 intermediate (int) tumors (*n* = 34 int-A, *n* = 12 int-B) and 16 malignant (mal) MGMs. The median age of patients at the time of first diagnosis was 61.0 years and the cohort displayed a female-to-male ratio of 1.8 to 1.0. To increase reliability and quantify rarer immune cell subsets, we used whole-tissue sections of newly diagnosed tumors with a median area of 20.00 mm^2^ (2.36 mm^2^–53.31 mm^2^). To assess immune infiltration rates, we quantified the number of CD20+ B cells and NKp46+ NK cells, respectively, relative to the TCC (Fig. 1b). This was similarly done using CD66b and EPX as markers for neutrophils (CD66b+ EPX- cells) and eosinophils (CD66b+ EPX+ cells; Fig. 1c). We observed that infiltration patterns of these immune cell subsets were highly variable across MGM specimens (Fig. 1d–g). Median NK cell infiltration was found to be 0.0063% and ranged from 0.00 to 1.65% (Fig. 1d). Frequencies of B cells were smaller and varied from 0.00% to 0.32% with a median infiltration of 0.0034% (Fig. 1e). Among the analyzed cell subsets, neutrophil infiltration was highest with a median infiltration of 0.0792% and differed widely from 0.00% to 12.00% (Fig. 1f), whereas eosinophil numbers were smallest with a median infiltration of 0.0023%, ranging from 0.00% to 0.17% (Fig. 1g). Notably, all analyzed tumors showed infiltration of at least one rarer immune cell population. However, a substantial number of newly diagnosed MGMs demonstrated no infiltration of NK cells (18.6%; 18/97), B cells (32.0%; 31/97) or eosinophils (33.7%, 31/92), while only 5.4% (5/92) of tumors displayed no neutrophil infiltration, suggesting an overall higher prevalence of neutrophils in newly diagnosed MGMs.

In summary, our tissue cytometry analysis revealed lower frequencies of B cells, NK cells, and eosinophils compared to neutrophils with overall heterogeneous infiltration patterns in newly diagnosed MGM specimens.

### Increased NK cell numbers are associated with a more benign behavior in meningioma

**Fig. 2:**
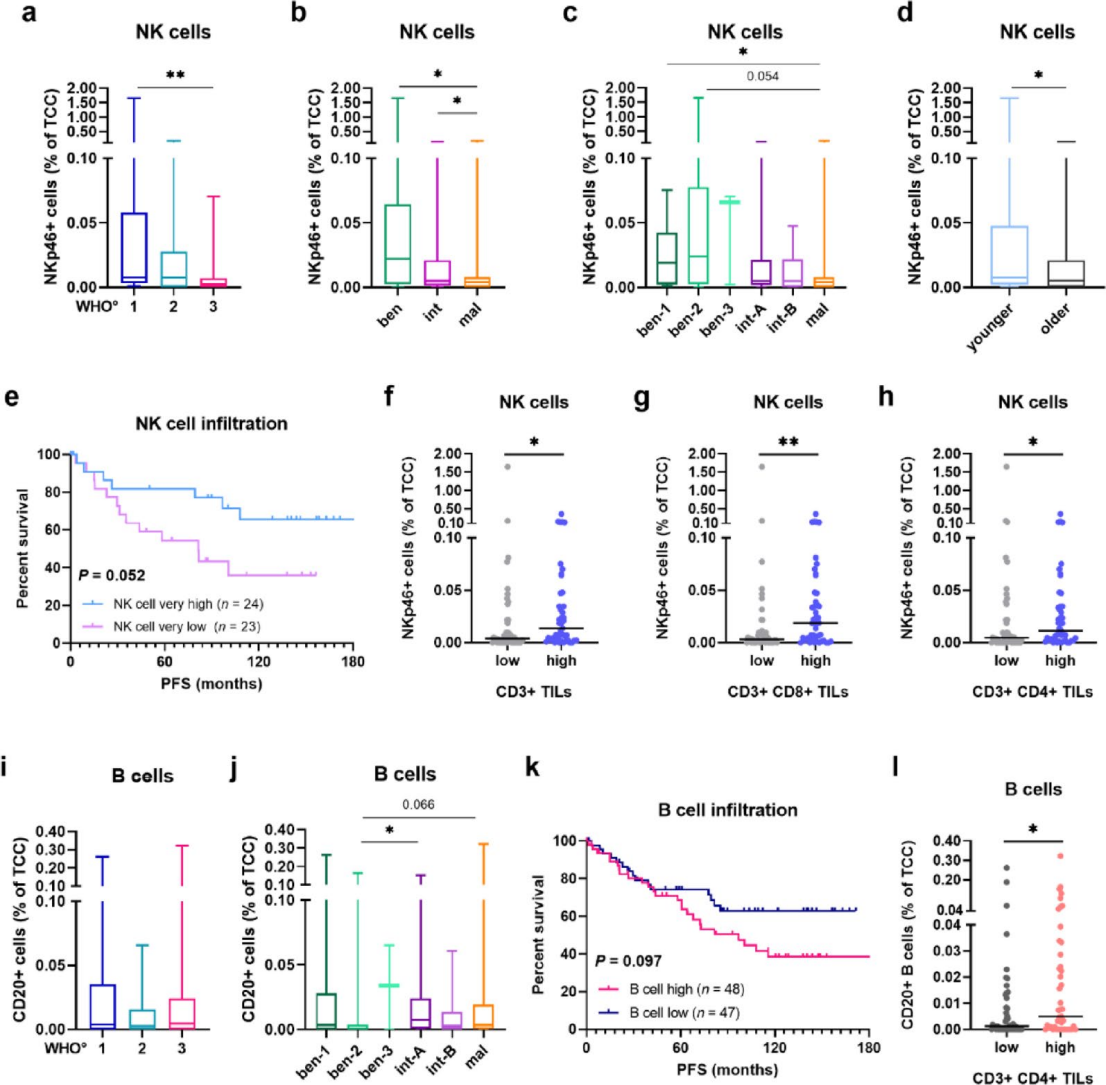
NK and B cell infiltration in newly diagnosed meningioma. **a**–**c** NK cell infiltration in newly diagnosed MGMs across **a** WHO grades, **b** methylation classes, **c** methylation subclasses. **d** NK cell infiltration of younger and older patients (median age of 61.0 years). **e** Kaplan–Meier plot for PFS based on quartile-split very high (light blue curve) and very low (purple curve) NK cell infiltration in newly diagnosed MGMs. **f**–**h** NK cell infiltration in patients with low or high infiltration of **f** total CD3+ TILs, **g** CD3+ CD8+ cytotoxic TILs, and **h** CD3+ CD4+ helper TILs. **i**–**j** B cell infiltration in newly diagnosed MGMs across **i** WHO grades, and **j** methylation subclasses. **k** Kaplan–Meier plot for PFS based on median-split high (pink curve) and low (dark blue curve) B cell infiltration in newly diagnosed MGMs. **l** B cell infiltration in patients with low or high infiltration of CD3+ CD4+ helper TILs. Statistical significance was calculated using Mann–Whitney-U test in (a-d, f-j, l), and log-rank test in (e, k). ben, benign; intermediate, int; mal, malignant; MGM, meningioma; NK, natural killer; PFS, progression-free survival; TCC, total cell count; TIL, tumor-infiltrating T lymphocyte. Statistical significance: *, *P* < 0.05; **, *P* < 0.01

Subsequently, we analyzed infiltration rates for each immune cell population in respect to clinical parameters, including WHO grades (1, 2, 3), methylation classes (ben, int, mal) and subclasses (ben-1/2/3, int-A/B, mal) as well as patient age and sex and states of prospective recurrency (minimum follow-up time of 60 months). For NK cells, median infiltration significantly decreased from WHO grade 1 (0.008%) to grade 3 tumors (0.003%; **, *P* < 0.01; Fig. 2a). This trend was even more pronounced regarding methylation classes, where NK cell frequencies were significantly reduced in malignant MGMs with a median of 0.004% compared to 0.005% in intermediate tumors (*, *P* < 0.05; Fig. 2b) and 0.022% in benign cases (*, *P* < 0.05; Fig. 2b). For methylation subclasses, a similar pattern was seen: NK cell rates declined from ben-1 and ben-2 tumors to malignant cases (*, *P* < 0.05 and *P* = 0.054, respectively; Fig. 2c), altogether suggesting a higher prevalence of NK cells in more benign MGMs. In addition, we found significantly higher NK cell frequencies in younger patients (below median age of 61.0 years; *, *P* < 0.05; Fig. 2d), while no sex-specific differences were observed (Suppl. Fig. S3a). When looking at prospective recurrency in newly diagnosed MGMs (i.e., patients experiencing a tumor recurrence within the follow-up time), NK cell levels tended to be higher in non-recurring (NR) tumors with a median infiltration of 0.0075% as compared to prospectively recurring (PR) cases with a median infiltration of 0.0051% (Suppl. Fig. S3b).

We then investigated whether NK cell infiltration influences the disease course in MGMs. To minimize bias in the PFS analysis, we included only patients who had undergone GTR (Simpson grade 1–3), had not received prior treatment, and whose follow-up period was at least 60 months. For the analysis, the patient cohort was then divided into low and high infiltration groups according to the median, or split into quartiles comparing the infiltration groups with highest or lowest infiltration, respectively. Survival analysis of the resulting patient cohort (*n* = 95) showed a tendency for improved outcome in tumors with high NK cell infiltration, but largely failed to reach a level of significance (*P* = 0.224, Suppl. Fig. S3c). However, this trend was much more pronounced in the quartile-based survival analysis. Patients with the highest NK cell infiltration (*n* = 24; light blue curve) demonstrated an almost significant survival advantage compared to patients with the lowest NK cell frequency (*n* = 23; purple curve; *P* = 0.052; Fig. 2e, Suppl. Fig. S3d).

Next, we studied whether NK cell infiltration is accompanied by the infiltration of T cells in newly diagnosed MGMs. To this end, we analyzed NK cell numbers in tumors with low or high T cell infiltration (split by the median) using our previously published data set on TILs in MGMs [34]. Interestingly, NK cell infiltration was significantly increased in newly diagnosed MGMs with high numbers of different T cell subsets including total CD3+ TILs (*, *P* < 0.05; Fig. 2f), cytotoxic CD3+ CD8+ TILs (**, *P* < 0.01; Fig. 2g), and CD3+ CD4+ TILs of a helper phenotype (*, *P* < 0.05; Fig. 2h), which could indicate a more immunostimulatory microenvironment in these tumors that ultimately leads to an improved patient outcome.

Altogether, our data showed that NK cells are more common in benign MGMs and that high NK cell infiltration is associated to higher infiltration of TILs.

### B cell infiltration tends to be higher in clinically aggressive meningioma

Subsequently, we performed similar analyses to assess B cell infiltration rates according to clinical features in newly diagnosed MGMs. In contrast to NK cells, we observed no significant differences in B cell numbers across WHO grades (Fig. 2i) or methylation classes (Suppl. Fig. S3e). Regarding methylation subclasses, B cell frequencies were significantly increased in int-A cases (*, *P* < 0.05), and also higher in malignant cases (*P* = 0.066; Fig. 2j), both compared to ben-2 tumors. In turn, we found no sex- and age-specific differences (Suppl. Fig. S3f-g) and across states of prospective recurrency, even though B cell rates were 3 × higher in PR tumors with a median infiltration of 0.0047% compared to NR tumors (0.0015%; Suppl. Fig. S3h).

We next analyzed survival outcomes (PFS) regarding B cell infiltration. For this purpose, we applied the above stated selection criteria (GTR, no prior treatment, follow-up > 60 months) in newly diagnosed MGMs and divided the resulting patient cohort into low and high infiltration groups according to the median. Although we observed no significant differences in survival outcomes for tumors with low or high B cell infiltration, patients’ tumors with high B cell numbers tended to be associated with lower survival rates (*P* = 0.097; Fig. 2k) in the long-term (> 60 months). In addition, we investigated whether B cell infiltration is also accompanied by T cell infiltration as described above [34]. Interestingly, we found that tumors with high numbers of T helper cells also had significantly increased B cell frequencies (*, *P* < 0.05; Fig. 2l), potentially highlighting their role as professional antigen-presenting cells (APCs) via major histocompatibility (MHC) class II (MHC-II) molecules. In turn, no significant differences in B cell frequencies were observed in relation to total TILs and cytotoxic TILs (Suppl. Fig. S3i-j).

Taken together, B cells appear to be less associated with clinical features of newly diagnosed MGMs and do not seem to play a significant prognostic role in disease progression and patient outcome most likely due to their overall low infiltration rates.

### Neutrophils and eosinophils demonstrate opposing prognostic roles in meningioma

**Fig. 3:**
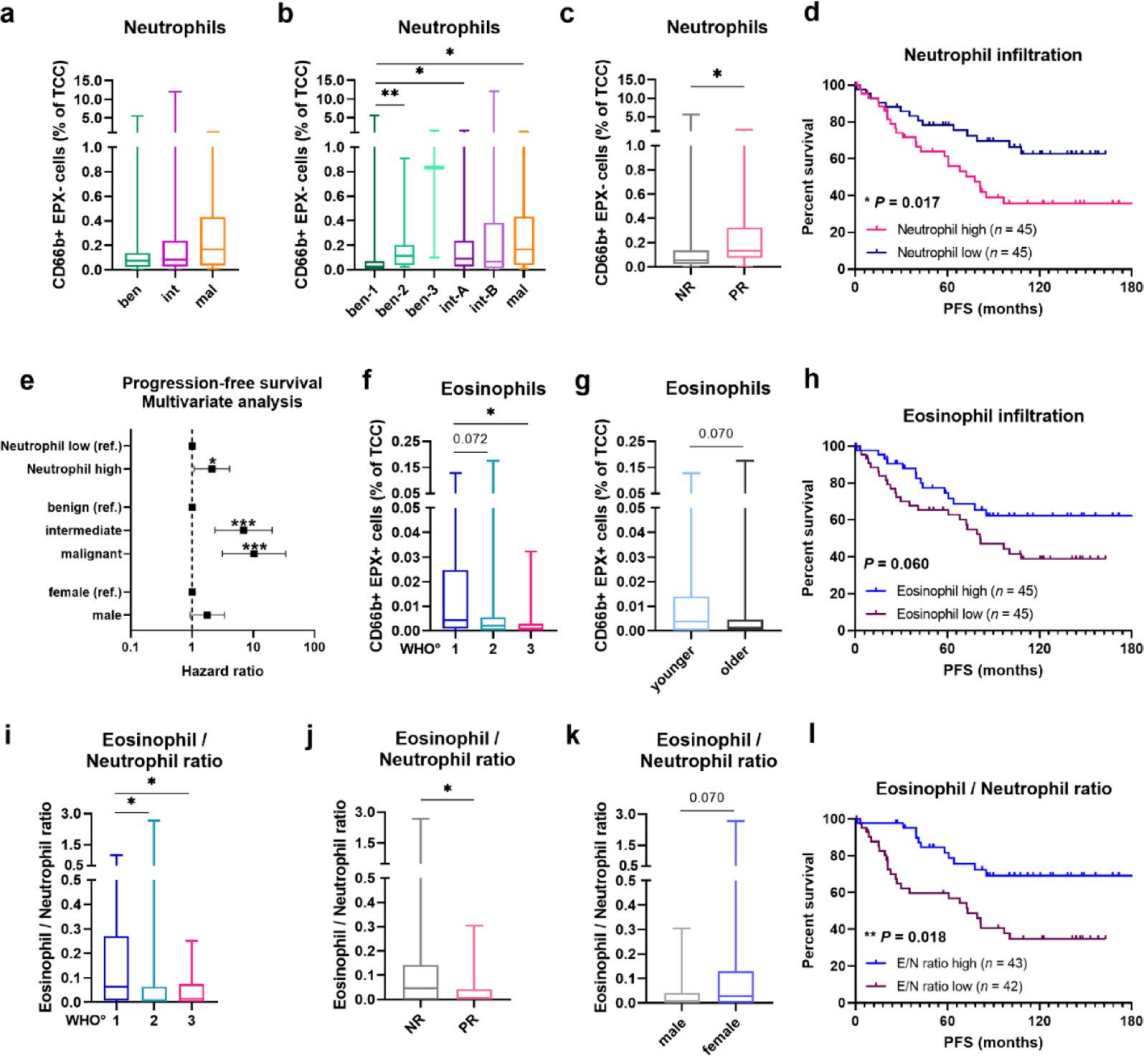
Neutrophil and eosinophil infiltration in newly diagnosed meningioma. **a** and **b** Neutrophil infiltration in newly diagnosed MGMs across **a** methylation classes, and **b** methylation subclasses. **c** Neutrophil infiltration in newly diagnosed MGMs including non-recurring (NR) and prospectively recurring (PR) tumors. **d** Kaplan–Meier plot for PFS based on median-split high (pink curve) and low (dark blue curve) neutrophil infiltration in newly diagnosed MGMs. **e** Multivariate survival analysis for neutrophil infiltration and PFS, including prognostic confounders (methylation classes and patient sex). **f**–**g** Eosinophil infiltration in newly diagnosed MGMs across **f** WHO grades, as well as **g** younger and older patients (median age of 61.0 years). **h** Kaplan–Meier plot for PFS based on median-split high (blue curve) and low (dark purple curve) eosinophil infiltration in newly diagnosed MGMs. **i**–**k** Ratio of eosinophil/neutrophil infiltration in newly diagnosed MGMs across **i** WHO grades, **j** recurrency states (NR, PR), and of **k** male and female patients. **l** Kaplan–Meier plot for PFS based on median-split high (blue curve) and low (dark purple curve) ratio of eosinophil/neutrophil infiltration in newly diagnosed MGMs. Five tumor samples had to be excluded for the E/N ratio due to values of zero in neutrophil infiltration. Statistical significance was calculated using Mann–Whitney-U test in (**a**–**c**, **f**–**g**, **i**–**k**), Cox proportional hazard model in (**e**) and log-rank test in (**d**, **h**, **l**). ben, benign; E/N, eosinophil-to-neutrophil; intermediate, int; mal, malignant; MGM, meningioma; NR, non-recurring; PFS, progression-free survival; PMR, prospectively malignant-recurring; PR, prospectively recurring; ref, reference; TCC, total cell count. Statistical significance: *, *P* < 0.05; **, *P* < 0.01; ***, *P* < 0.001

Likewise, we assessed neutrophil and eosinophil infiltration in association with clinical parameters in newly diagnosed MGMs. Even though neutrophil frequencies displayed no substantial differences across WHO grades, patient sex and age groups (Suppl. Fig. S4a–c) as well as methylation classes (Fig. 3a), we observed significant differences across methylation subclasses and an overall tendency towards higher neutrophil rates in clinically aggressive tumors as defined by methylation analysis (Fig. 3b). In comparison to ben-1 MGMs, neutrophil numbers were significantly elevated in ben-2 tumors (**, *P* < 0.01), int-A cases (*, *P* < 0.05) and malignant MGMs (*, *P* < 0.05), showing a 6.4-fold increase as compared to ben-1 tumors with a median infiltration of 0.0259% to 0.166% in malignant ones. Additionally, neutrophil infiltration was significantly increased in PR tumors compared to NR tumors (*, *P* < 0.05; Fig. 3c). Furthermore, analyses of the survival cohort (*n* = 90) revealed that high neutrophil infiltration (median-based split) is associated with inferior PFS in patients with newly diagnosed MGMs (selection criteria as stated above; *, *P* < 0.05; Fig. 3d), suggesting an unfavorable prognostic role of this granulocyte subset. Subsequently, a multivariate analysis was performed, including methylation classes and patient sex as relevant prognostic confounders. Importantly, this analysis revealed that high neutrophil infiltration (median split) is an independent prognostic factor for inferior PFS in patients with newly diagnosed MGMs (hazard ratio (HR) = 2.11; *P* = 0.03; Fig. 3e, Suppl. Table S1).

Next, we performed similar analyses for eosinophils. In the study cohort, eosinophil infiltration tended to be decreased in clinically aggressive tumors and was significantly lower in WHO grade 3 tumors compared to grade 1 MGMs (*, *P* < 0.05; Fig. 3f). However, we observed no substantial differences between methylation classes and subclasses (Suppl. Fig. S4d–e), male and female patients (Suppl. Fig. S4f) as well as states of prospective recurrency even though eosinophils were higher in NR tumors as compared to PR MGMs (*P* = 0.079, Suppl. Fig. S4g). Further, eosinophil frequencies were higher in younger patients (< 61.0 years), however also without reaching a level of significance (*P* = 0.07; Fig. 3g). We then performed survival analyses with the selection criteria as stated above for newly diagnosed MGMs (*n* = 90). Interestingly, we observed a survival benefit for tumors with high eosinophil infiltration, but the analysis did not reach a level of significance (*P* = 0.06; Fig. 3h). Likewise, the multivariate survival analysis demonstrated no significant associations (data not shown).

As eosinophils and neutrophils appeared to have opposing prognostic roles in newly diagnosed MGMs, we next analyzed the eosinophil-to-neutrophil (E/N) ratio by dividing determined eosinophil numbers by the neutrophil numbers in association with clinical features and survival outcomes. Regarding WHO grades, we observed a significantly higher E/N ratio in WHO grade 1 MGMs compared to higher-grade tumors (*, *P* < 0.05; Fig. 3i). The same tendency was seen for methylation classes and subclasses but without significant differences between groups (Suppl. Fig. S4h-i). No age-specific differences were monitored (Suppl. Fig. S4j), whereas we found a higher E/N ratio in female patients (*P* = 0.07; Fig. 3k). In turn, when analyzing survival differences in the resulting cohort (*n* = 85, selection criteria as stated above but excluding neutrophil cases with values of 0), patients of tumors with a high E/N ratio demonstrated a significant survival benefit compared to the ones with a low E/N ratio (**, *P* < 0.01; Fig. 3l).

In summary, neutrophils and eosinophils appear to exhibit opposing infiltration patterns in newly diagnosed MGMs, ultimately leading to a more favorable prognosis for patients whose tumors show lower neutrophil numbers but higher rates of eosinophils as well as an overall higher E/N ratio.

### Integrative analysis identifies five meningioma immune ecotypes

To comprehensively analyze the immunological composition in newly diagnosed MGM, we integrated our previously published TIL [34] and TAM [21] data sets. We first performed a correlation analysis of immune cell infiltration data that revealed a significant correlation between NK and T cell infiltration as well as neutrophils and eosinophils (Suppl. Fig S5a). Secondly, we inferred CNVs from DNA methylation data and analyzed chromosomal arm losses in association with immune cell infiltration, discovering significant associations for chromosomal arms 1p, 10q and 22q (Suppl. Fig S5b-d). Interestingly, infiltration of CD3+ TILs, CD4+ TILs and CD8+ TILs was significantly lower in newly diagnosed MGMs with chromosome 1p loss (Suppl. Fig. S5b), which was previously associated with an increased risk of tumor recurrence [24]. Additionally, we found that infiltration of B cells is significantly lower in tumors with prognostically unfavorable chromosome 10q and 22q loss (Suppl. Fig. S5c–d, [28, 30]).

**Fig. 4:**
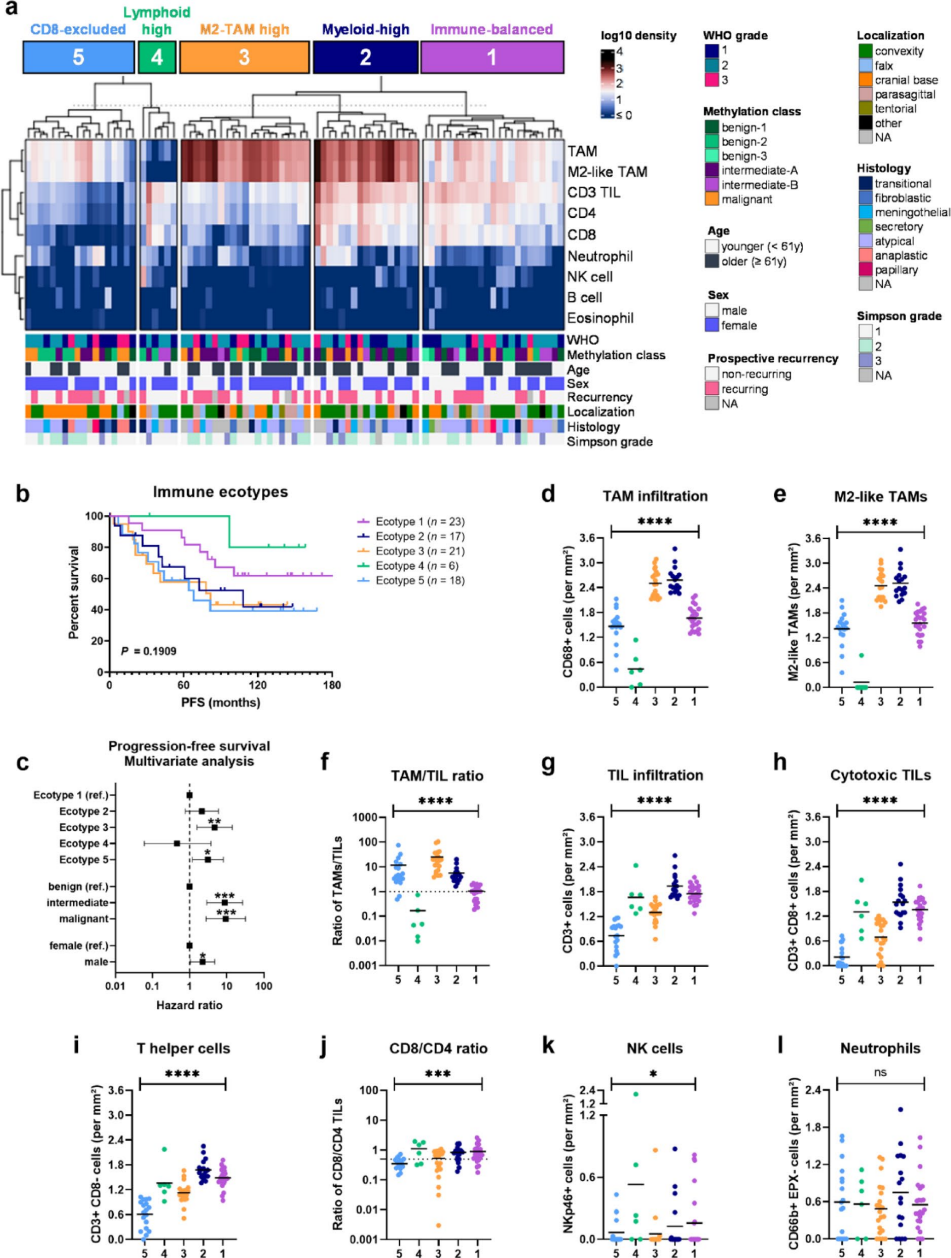
Immune ecotypes in newly-diagnosed meningioma. **a** Unbiased consensus clustering of log10-transformed cell densities (cells/mm^2^) quantified in *n* = 85 newly diagnosed MGM by tissue cytometry, heatmap with clinical parameters. **b** Kaplan–Meier plot for PFS based on immune ecotypes defined in (**a)** in newly diagnosed MGMs **c** Multivariate survival analysis for immune ecotypes and PFS, including prognostic confounders (methylation classes and patient sex). **d**–**l** Log10-transformed cell densities by immune ecotype showing **d** TAM infiltration, **e** M2-like TAM infiltration, **f** ratio of TAM/TIL densities, **g** TIL infiltration, **h** cytotoxic TIL infiltration, **i** T helper cell infiltration, **j** ratio of CD8/CD4 TIL densities, **k** NK cell infiltration, and **l** neutrophil infiltration. Statistical significance was calculated using log-rank test in (**b**), Cox proportional hazard model in (**c**) and ordinary one-way ANOVA tests in (**f**–**l**). MGM, meningioma; NA, not available; NK, natural killer; NR, non-recurring; PFS, progression-free survival; PR, prospectively recurring; ref, reference; TAM, tumor-associated macrophage; TIL, tumor-infiltrating T lymphocyte. Statistical significance: *, *P* < 0.05; **, *P* < 0.01; ***, *P* < 0.001; ****, *P* < 0.0001

Furthermore, we performed unbiased consensus clustering of immune cell densities (per mm^2^) in each tumor specimen across the cohort (*n* = 85; Fig. 4a–c, Suppl. Fig. S6a–e). This integrative approach identified five MGM-specific immune ecotypes with differential survival outcomes and immune cell infiltration characteristics (Fig. 4b–l, Suppl. Fig. S6c–e). These immune ecotypes were overall dominated by TAM and TIL infiltration patterns but were also informative on other rarer immune cell subsets such as neutrophils and NK cells. Over one quarter (27%, *n* = 23) of cases were categorized into ecotype 1 (purple), designated as “immune-balanced” and showing an intermediate patient outcome with a median PFS of 103.6 months. Ecotype 1 was particularly characterized by a lower TAM and higher TIL infiltration, resulting in a mean TAM/TIL ratio of 1.0, and was also characterized by higher NK cell frequencies. In turn, ecotype 2 (20%, *n* = 17) termed as “myeloid high” (dark blue) showed the overall highest infiltration of TAMs, immunosuppressive M2-like TAMs as well as neutrophils and displayed poor patient outcome with a median PFS of 60.7 months. For ecotype 3 (25%, *n* = 21), referred to as M2-TAM high (orange), we observed the second highest infiltration of TAMs and immunosuppressive M2-like TAMs and moreover the highest TAM/TIL ratio (mean of 24.9), suggesting a TME that is essentially dominated by immunosuppressive TAMs in this ecotype. This TAM dominance was further mirrored in the most inferior survival outcome of MGM patients showing a median PFS of only 41.0 months for ecotype 3. In contrast, ecotype 4 (green) exhibited the most superior patient outcome with a median PFS of 135.0 months, but consisted only of *n* = 6 cases (7%). Nevertheless, this ecotype was especially characterized by mostly absent TAMs and M2-like TAMs while densities of TILs were substantially higher, which resulted in the only negative TAM/TIL ratio and thus highest TIL/TAM ratio (mean of 37.2), indicating a TIL dominance in this ecotype. Since the NK cell density was also highest in ecotype 4, this was therefore described as "lymphoid-high". For ecotype 5 (light blue), including *n* = 18 (21%) MGM cases, we observed poor survival rates with a median PFS of 66.1 months comparable to the patient outcomes of ecotypes 2 and 3, both of which are TAM-dominated. Interestingly, ecotype 5 showed not only low frequencies of TAMs and M2-like TAMs but also lower numbers of cytotoxic CD8+ TILs as well as higher numbers of CD4+ T helper cells, resulting in the lowest CD8/CD4 ratio (mean of 0.35) and was therefore designated as “CD8-excluded”. Additionally, we observed higher neutrophil counts, which might support an immunosuppressive tumor milieu. Even though the univariate analysis showed no significant differences in survival for the immune ecotypes, we performed multivariate PFS analysis including prognostic confounders, such as methylation classes and patient sex. Importantly, this analysis demonstrated that immune ecotypes 3 and 5 were independent predictors of worse PFS (ecotype 3: HR = 4.71, *P* = 0.006; ecotype 5: HR = 3.09, *P* = 0.022; Fig. 4c, Suppl. Table S2) in newly diagnosed MGMs. These findings emphasize the deleterious prognostic impact of high immunosuppressive M2-like TAM levels as well as low numbers of cytotoxic TILs being characteristic for both unfavorable ecotypes.

Subsequently, we investigated potential associations of the MGM-specific immune ecotypes with several MGM-relevant clinicopathological features, including WHO grades, methylation (sub)classes, patient age and sex, anatomical location, histological subtypes and states of prospective recurrency (Suppl. Fig. S6f-l). However, these analyses revealed no significant associations but were accompanied by some interesting observations. For example, the prognostically most favorable “lymphoid-high” ecotype 4, consisting only of WHO grade 1 and 2 tumors, demonstrated a higher proportion of younger age and female patients (each with a proportion of 80%). In turn, “M2-TAM high” ecotype 3 identified as the prognostically most unfavorable category contained the highest proportion of higher-grade tumors (76.2% WHO grade 2 and 3) and older patients (66.7%). Interestingly, ecotype 5 that was characterized by CD8 exclusion and poor survival outcome encompassed a major proportion of MGM localized in the cranial base (55.6%). Further, we observed the lowest proportion of benign tumors (29.4%) in the “myeloid-high” ecotype 2, which also displayed poor survival rates. Additionally, the “immune-balanced” ecotype 1 showed intermediate patient outcome even though it contained a high number of clinically aggressive tumors (69.6% WHO grade 2 and 3), suggesting a favorable prognostic impact of the TIL over TAM balance.

Taken together, our cell type-integrative analysis unraveled five MGM immune ecotypes with distinct immune cell infiltration patterns. Noteworthy, immune ecotypes showed differential survival outcomes in MGM patients and ecotypes 3 and 5 were identified as independent prognostic factors of inferior PFS. Therefore, this analysis suggests a diverse immune landscape in newly diagnosed MGMs that might ultimately affect patient prognosis and treatment.

## Discussion

MGMs are often considered “immunologically cold” brain tumors due to their low tumor mutational burden and low lymphocyte numbers [42, 43]. This notion has been further reinforced by the limited efficacy observed in clinical trials on T cell-directed anti-PD1 therapies, such as pembrolizumab and nivolumab, where only a small subset of patients appeared to derive benefit [3, 5]. Nevertheless, recent studies on immune infiltrates have described significant prognostic roles for TILs and TAMs in MGM [21, 34]. These findings warrant further investigations into the tumor’s immunological landscape and its impact on disease progression in order to ultimately design effective anti-cancer immunotherapies.

To address this need, we examined the infiltration of additional immune cell subsets by tissue cytometry in a large and clinically well-annotated cohort of *n* = 97 newly diagnosed MGMs. We investigated in detail the frequencies of B cells, NK cells, neutrophils and eosinophils in association with a number of clinicopathological parameters including WHO grading, methylation classes, patient age and sex, anatomical locations, histological subtypes as well as clinical outcomes. Moreover, we integrated our previously published TAM and TIL data and applied a cell type-integrative approach to unravel immune ecotypes in a final cohort of *n* = 85 newly diagnosed MGM. Overall, our analyses revealed that the infiltration rates of B cells, NK cells, eosinophils and neutrophils varied widely and were rather low (below 0.1% of TCC), which is in line with previous investigations [8, 10, 12, 44]. Among the analyzed cell subsets, neutrophils showed the highest median infiltration and were present in almost 95% of tumors, whereas B cells and eosinophils were absent in one third of tissue specimens. Interestingly, higher NK cell infiltration was associated with higher TIL infiltration, which could indicate a more immunostimulatory TME in these MGMs with elevated numbers of immune effector cells.

Compared to previous studies [8, 10, 12, 44], a major strength of this work is its focus on disease progression and prognostic implications. To this end, our study cohort included a significant frequency of higher-grade tumors, with 49 WHO grade 2 tumors and 17 WHO grade 3 MGMs. Moreover, patients had a median follow-up time of 86.6 months, and only those who met stringent selection criteria (GTR, no prior treatment, and follow-up > 60 months) were included in the survival analysis to reduce a bias caused by clinical confounders. This approach allowed us to examine the prognostic significance of the analyzed immune cell types. Importantly, despite their overall relatively low infiltration rates, elevated neutrophil levels were significantly linked to poor patient outcome and were also identified as an independent predictor for poor survival. Notably, a previous study by Karimi and colleagues analyzed peripheral blood samples from *n* = 222 MGM patients and identified preoperative blood neutrophilia as an independent prognostic marker for tumor recurrence [16]. Even though there is no clear association between systemic blood neutrophilia and presence of tumor-associated neutrophils (TANs) [18], these data emphasize an unfavorable prognostic role of neutrophils in MGM. In other brain malignancies, Maas and colleagues reported significant frequencies of neutrophils with higher rates in human brain metastases (BrMs) of breast, lung and other cancer types compared to *IDH* wildtype and mutant gliomas. Further, the authors showed that TANs present an immunosuppressive capacity, which also suggests a negative impact of neutrophils on brain tumor progression [23]. Moreover, in multiple cancer mouse models, a link between neutrophil infiltration and tumor necrosis has been demonstrated: A recent study by Adrover and colleagues identified a neutrophil subset capable of triggering pleomorphic tumor necrosis via neutrophil extracellular trap (NET)–driven occlusion of the tumor vasculature, suggesting tumor necrosis to be an active process that is mediated by a distinct vascular-restricted neutrophil population [1, 11]. However, in our study, we found no link between high neutrophil infiltration and tumor necrosis in MGM as necrotic areas were largely excluded from the tissue cytometry analysis.

For eosinophils, other studies described an inverse association between glioblastoma risk and atopic diseases such as allergic conditions where eosinophils are highly prevalent [9, 38]. Further, a study by Blomberg and colleagues observed an increase in systemic and intratumoral eosinophils in metastatic breast cancer patients and mouse models of breast cancer responding to ICB treatment [4], suggesting altogether a beneficial prognostic role of this granulocyte subset. These observations are in accordance with our data linking higher eosinophil-to-neutrophil ratio to improved PFS in MGM patients. Regarding lymphocytes, a study by Klemm and colleagues reported lower frequencies of B and NK cells in primary brain tumors compared to BrMs of different primary cancers (breast, lung, melanoma) and overall lower numbers as compared to TILs [17], which has also been described for MGM in this study and before [10, 12, 34, 39, 44]. Instead, we found that lower numbers of B cells were associated with prognostically unfavorable losses in chromosome 10q and 22q [28, 30]. In line with this observation, a study by Cakmak and colleagues identified a subset of gliomas with tertiary lymphoid structures (TLS), whose presence was associated with prolonged overall survival in patients [7]. Moreover, a recent study by Mughal et al. reported similar findings regarding the presence of TLS in BrMs of different primary cancers, with TLS also proving to be a prognostically favorable feature of the immune landscape for BrMs originating from lung cancer [27]. To the best of our knowledge, TLS have not yet been described in MGM. As we have not stained tumor specimens specifically for TLS in our cohort, including markers for T cells, B cells, dendritic cells, and high endothelial venules [36], this will be the subject of future investigations.

Noteworthy, an important pillar of our study is the integrative ecotype analysis where we analyzed the cell densities of TAMs, immunosuppressive M2-like TAMs, TIL subsets (CD3, CD8, CD4), NK cells, B lymphocytes, neutrophils and eosinophils by unbiased consensus clustering. Using this approach, we identified five MGM-specific immune ecotypes in newly diagnosed tumors, which displayed diverse immune cell infiltration patterns and differential survival outcomes. This is of particular interest as our findings suggest that MGMs exhibit diverse and complex immunological landscapes with prognostic implications. “Immune-balanced” ecotype 1 and “lymphoid-high” ecotype 4 showed both higher TIL and NK cell densities as well as lower numbers of immunosuppressive TAMs and were associated with improved patient outcome. In turn, TAM-dominated ecotypes 2 and 3 (“myeloid-high” and “M2-TAM high”) exhibited the most inferior patient outcomes and were accompanied by either high neutrophil levels or lower T cell counts. Interestingly, ecotype 5 was characterized by low TAM frequencies, and showed a significant enrichment of CD4 TILs compared to CD8 TILs, the latter of which were almost absent in these tumors. This CD8 exclusion was further mirrored in a similarly poor outcome to that of the TAM-dominated ecotypes 2 and 3. Even though our univariate survival analysis failed to reach a level of significance, most likely due to the low sample size in each subset, the multivariate analysis identified ecotype 3 and 5 as independent predictors of inferior PFS in MGM patients. These results corroborate our previous studies on the opposing prognostic roles of TAMs and TILs in MGM [21, 34], and highlight the importance of distinguishing between anti-tumor and tumor-supportive immune cell subtypes, as well as their ratio. However, future functional studies must answer the question of whether these rare cell types and the identified MGM-specific immune ecotypes are merely markers for better patient outcomes or actually mediators with functional consequences.

Nevertheless, a comprehensive immune profiling of MGMs might enable better patient stratification in immunomodulatory clinical trials to enhance objective response rates. Furthermore, (adjuvant) radiotherapy could help to convert immunologically cold or CD8-excluded immune desert tumors (ecotypes 2, 3 and 5) into immunologically hot MGMs (ecotypes 1 and 4) with higher T cell infiltration. In addition, radiation could be combined with ICB therapies targeting TILs, other immunotherapies aiming at TAM reprogramming, such as CSF1R blockade [2, 13–15, 22, 44], novel therapeutic strategies to target neutrophils [19], or even NK cells, such as IL15R agonists to enhance anti-tumor immunity [29].

One major limitation of our study with regard to routine clinical practice is that we used fresh-frozen tumor tissue samples and acetone-fixed cryosections, as tumor diagnostics in pathology are performed with formalin-fixed, paraffin-embedded (FFPE) tissue blocks. However, since we used common markers for characterizing immune cells and numerous other studies have already analyzed immune cell infiltration in MGM using FFPE tissue [10, 12, 33, 44, 46], this should allow for immunotyping with FFPE tissue samples analogous to our workflow.

In summary, in this study we analyzed rarer immune cell populations in newly diagnosed MGMs, including B lymphocytes, NK cells, neutrophils and eosinophils and found substantial associations with survival and chromosomal alterations in MGM patients. Moreover, our integrative analysis unraveled five MGM-specific immune ecotypes that are characterized by diverse immune infiltration patterns, of which ecotype 3 and 5 are associated with inferior survival outcomes. Therefore, this work provides a framework for future investigations into the immune landscape of MGM and will hopefully support patient stratification in future clinical trials of immunotherapies based on the patient's individual immune ecotype.

## Supplementary Information

Below is the link to the electronic supplementary material.

Supplementary Material 1.

## Data Availability

All data are being securely held within the Division of Experimental Neurosurgery, Department of Neurosurgery at the University Hospital of Heidelberg, Germany. All data have been systematically cataloged and are readily available from the corresponding author (CHM) upon reasonable request.

## Abbreviations

APC: Antigen-presenting cell
ben: Benign
BrM: Brain metastasis
CDF: Cumulative distribution function
cluserAlg: Clustering algorithm
CNS: Central nervous system
CNV: Copy number variation
E/N: Eosinophil-to-neutrophil
EPX: Eosinophil peroxidase
FFPE: Formalin-fixed, paraffin-embedded
GTR: Gross total resection
HR: Hazard ratio
ICB: Immune checkpoint blockade
int: Intermediate
mal: Malignant
maxK: Maximum number of clusters
MDSC: Myeloid-derived suppressor cells
MGM: Meningioma
MHC: Major histocompatibility
MIA: Multi-image alignment
NA: Not available
NET: Neutrophil extracellular trap
NK: Natural killer
NR: Non-recurring
OS: Overall survival
pFeature: Feature resampling proportion
PFS: Progression-free survival
pItem: Item resampling proportion
pr: Prospectively recurring
reps: Number of resampling iterations
ROI: Region of interest
TAM: Tumor-associated macrophages
TAN: Tumor-associated neutrophil
TCC: Total cell count
TIL: Tumor-infiltrating T lymphocytes
TLS: Tertiary lymphoid structures
WHO: World health organization
