# Supplementary material for: Profiling of rare immune cell populations and integrative analysis identify immune ecotypes in newly diagnosed meningiomas
Source: Acta Neuropathol Commun. 2026 Mar 18;14:85. doi: 10.1186/s40478-026-02276-0 (PMC13063855; doi:10.1186/s40478-026-02276-0)
Supplement: Supplementary file 1 — Supplementary Material 1. [file 40478_2026_2276_MOESM1_ESM.pdf]

SUPPLEMENTARY MATERIAL

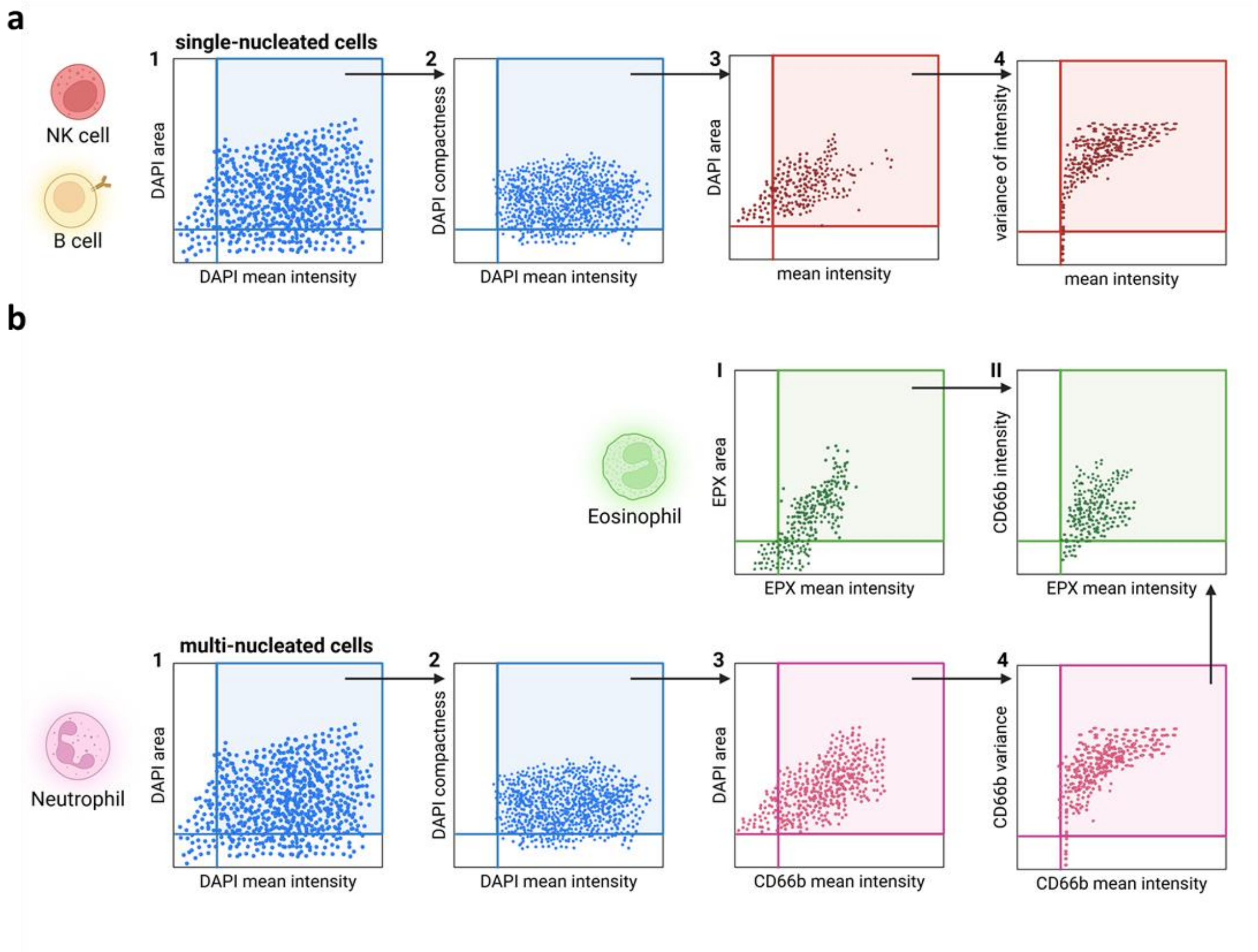

Suppl. Figure S1: Gating scheme for tissue cytometry analysis.

a Exemplary schematic gating sequence for NK cells (and B cells, respectively). Analysis of 1. DAPI mean intensity (x-axis) and DAPI area (y-axis), and 2. DAPI mean intensity (x-axis) and DAPI compactness (y-axis) to quantify all single-nucleated cells, followed by analysis of 3. NKp46 (or CD20) mean intensity (x-axis) and DAPI area (y-axis) with 4. fine-tuning analysis of NKp46 (or CD20) mean intensity (x-axis) and NKp46 (or CD20) variance of intensity (y-axis) to exclude unspecific staining and artifacts. Double positive cells for NKp46 and CD20 cells were subsequently excluded. **b** Exemplary schematic gating sequence for neutrophils and eosinophils. Nuclei detection for granulocytes was modified to detect large multi-segmented nuclei: Analysis of 1. DAPI mean intensity (x-axis) and DAPI area (y-axis), and 2. DAPI mean intensity (x-axis) and DAPI compactness (y-axis) to quantify all multi-nucleated cells, followed by analysis of 3. CD66b mean intensity (x-axis) and DAPI area (y-axis) with 4. fine-tuning analysis of CD66b mean intensity (x-axis) and CD66b variance of intensity (y-axis) to exclude unspecific staining and artifacts. As the EPX staining was more peripheral and extended to the vicinity of adjacent nuclei, the nuclear detection was first disabled to avoid missed detections of the scarce eosinophils. Analysis of eosinophils by I. EPX mean intensity (x-axis) and EPX area (y-axis), and II. EPX mean intensity (x-axis), and CD66b+ cells (CD66b mean intensity (y-axis)). Consequently, neutrophils were identified as CD66b+ EPX- cells and eosinophils as CD66b+ EPX+ cells.

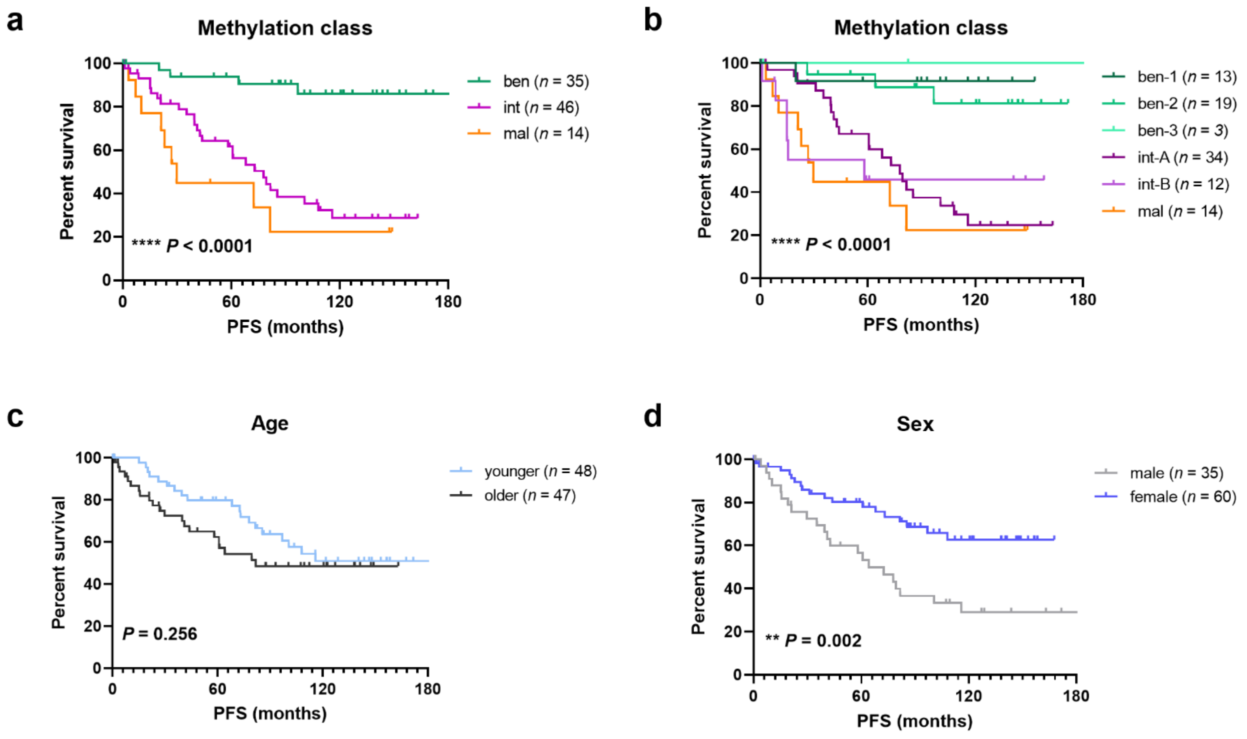

Suppl. Figure S2: Survival analysis of methylation classes, patient age and sex in newly diagnosed meningioma.

a-d Kaplan-Meier plot for PFS based on (**a**) methylation classes, (**b**) methylation subclasses, (**c**) patient age (based on median age of 61.0y), and (**d**) patient sex in newly diagnosed MGMs. Statistical significance was calculated using log-rank test. ben, benign; int, intermediate; mal, malignant; MGM, meningioma; PFS, progression-free survival; y, years. Statistical significance: **, *P*<0.01, ****, *P*<0.0001.

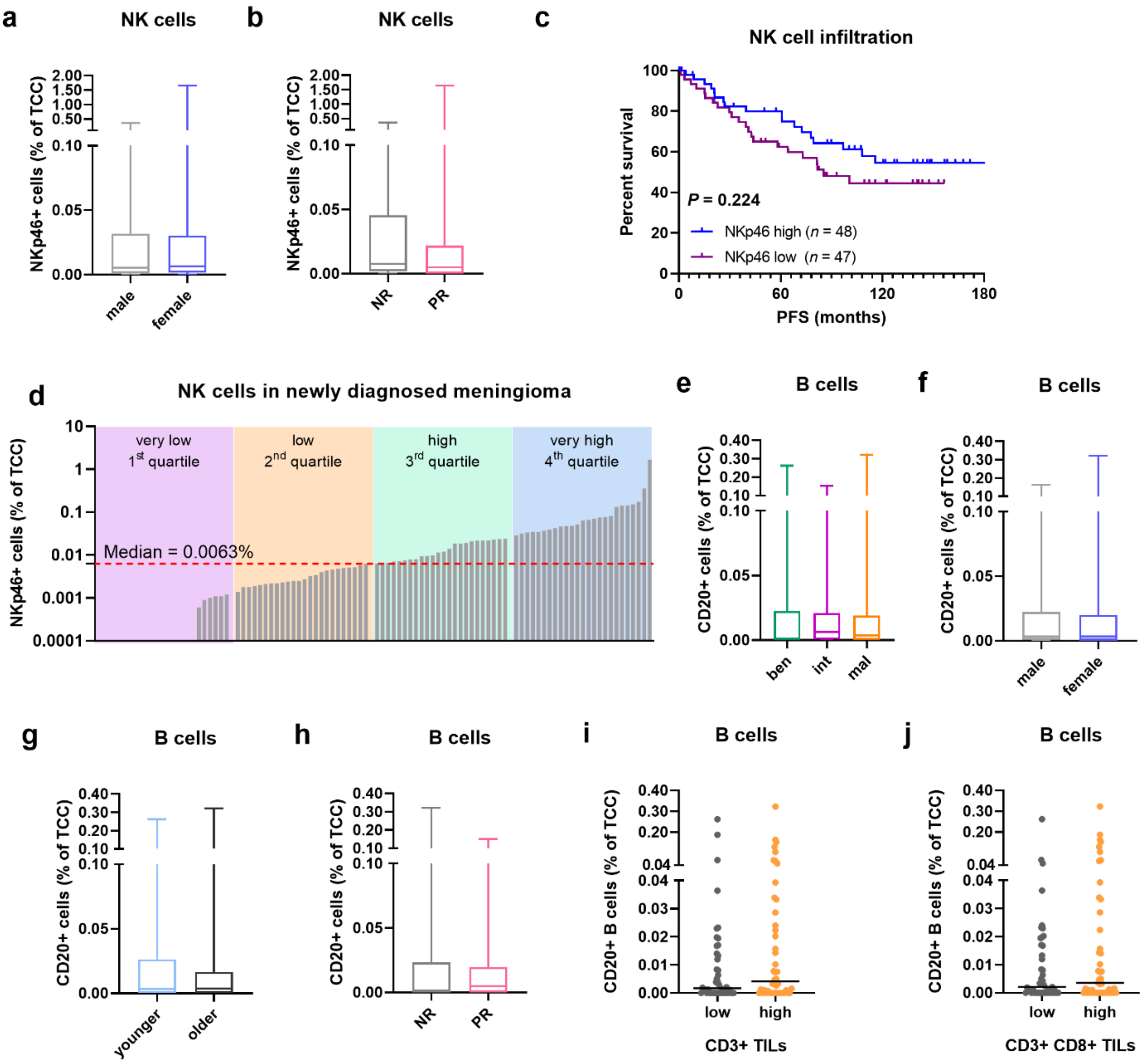

Suppl. Figure S3: NK cell and B cell infiltration in newly diagnosed meningioma.

a NK cell infiltration in newly diagnosed MGMs of female and male patients. **b** NK cell infiltration in newly diagnosed MGMs including non-recurring (NR) and prospectively recurring (PR) tumors. **c** Kaplan-Meier plot for PFS based on median-split high (blue curve) and low (purple curve) NK cell infiltration in newly diagnosed MGMs. **d** NK cell infiltration in newly diagnosed MGM showing quartile separation. **e** B cell infiltration in newly diagnosed MGMs across methylation classes. **f-h** B cell infiltration in newly diagnosed MGMs of (**f**) male and female patients, (**g**) younger and older patients (median age of 61.0 years), and across (**h**) recurrency states (NR, PR). **i-j** B cell infiltration in patients with low or high infiltration of (**i**) CD3+ TILs and of (**j**) CD3+ CD8+ cytotoxic TILs. Statistical significance was calculated using Mann-Whitney-U test in (a-b, e-j), and log-rank test in (d). ben, benign; intermediate, int; mal, malignant; MGM, meningioma; NK, natural killer; NR, non-recurring; PFS, progression-free survival; PR, prospectively recurring TCC, total cell count; TIL, tumor-infiltrating T lymphocyte.

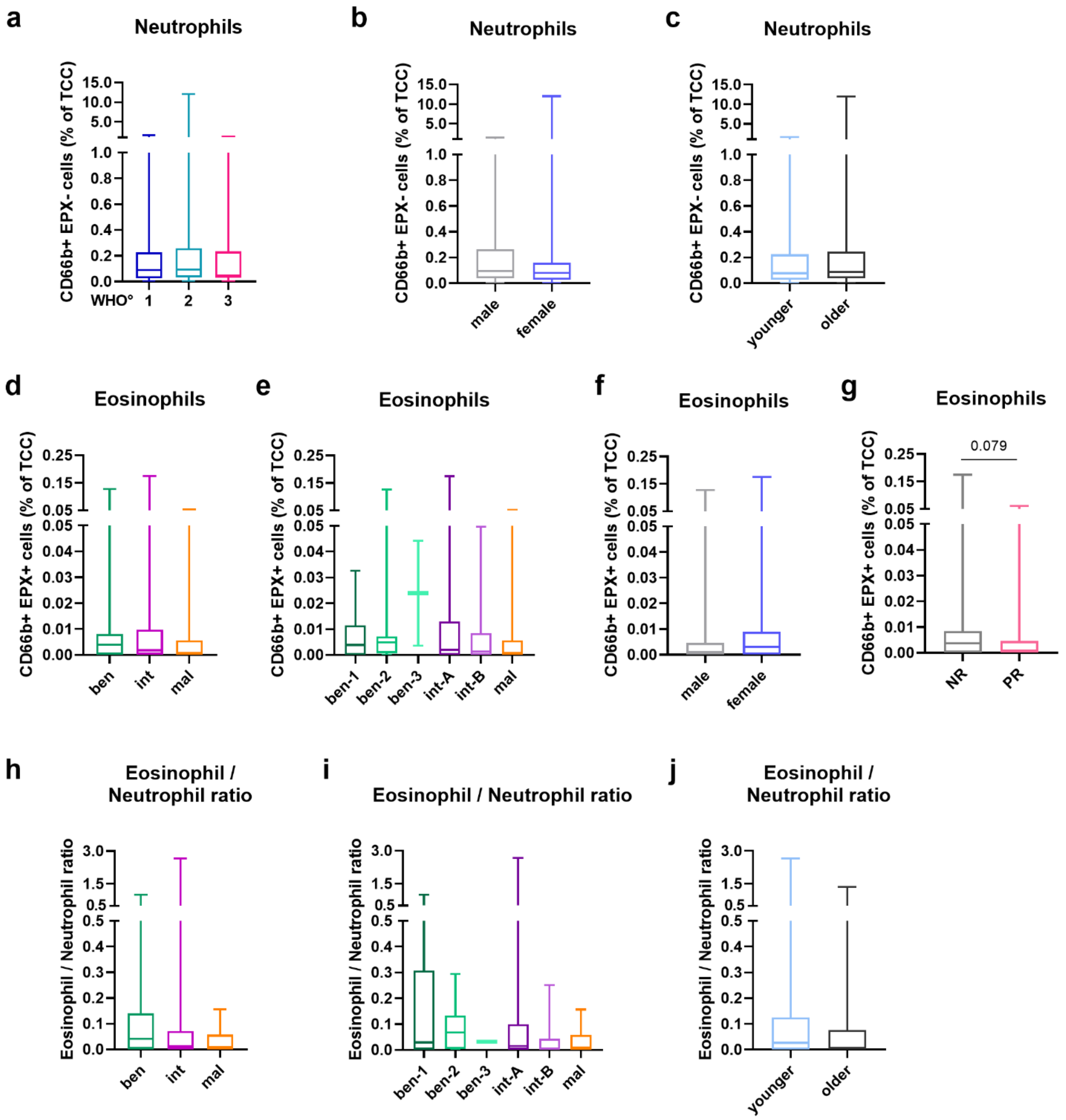

Suppl. Figure S4: Neutrophil and eosinophil infiltration in newly diagnosed meningioma.

a-c Neutrophil infiltration in newly diagnosed MGMs across (**a**) WHO grades, of (**b**) female and male patients, and (c) younger and older patients (median age of 61.0 years). **d-f** Eosinophil infiltration in newly diagnosed MGMs across (**d**) methylation classes, (**e**) methylation subclasses, and of (**f**) female and male patients. **g** Eosinophil infiltration in newly diagnosed MGMs including non-recurring (NR), and prospectively recurring (PR) tumors. **h-i** Ratio of eosinophil/neutrophil infiltration in newly diagnosed MGMs across (**h**) methylation classes, (**i**) methylation subclasses, and of (**j**) younger and older patients (median age of 61.0 years). Statistical significance was calculated using Mann-Whitney-U test. ben, benign; intermediate, int; mal, malignant; MGM, meningioma; NR, non-recurring; PR, prospectively recurring TCC, total cell count.

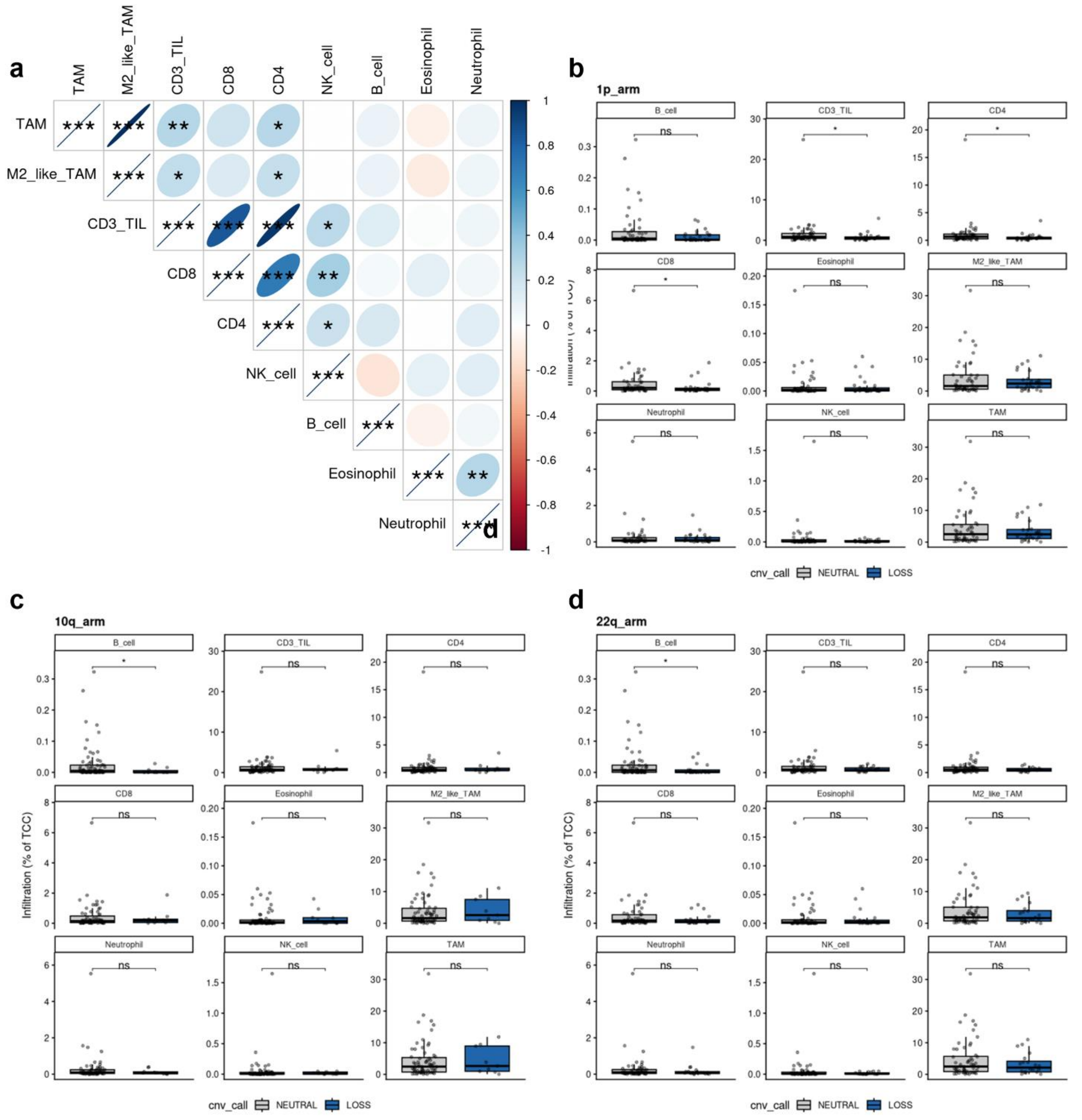

Suppl. Figure S5: Correlation analysis of immune cell infiltration data and analysis of CNV calls in newly diagnosed meningioma.

a Correlation matrix of immune cell infiltration numbers ordered by Spearman correlation. **b-d** Infiltration data (% of TCC) for immune cell types in association with CNV calls showing chromosomal arms (**b**) 1p, (**c**), 10q, and (**d**) 22q. Statistical significance was calculated using Spearman correlation in (a), and Mann–Whitney-U tests in (b-d). CNV, copy number variation; NK, natural killer cell; TAM, tumor-associated macrophage; TCC, total cell count; TIL, tumor-infiltrating T lymphocyte. Statistical significance: *, P<0.05; **, P<0.01, ***, P<0.001.

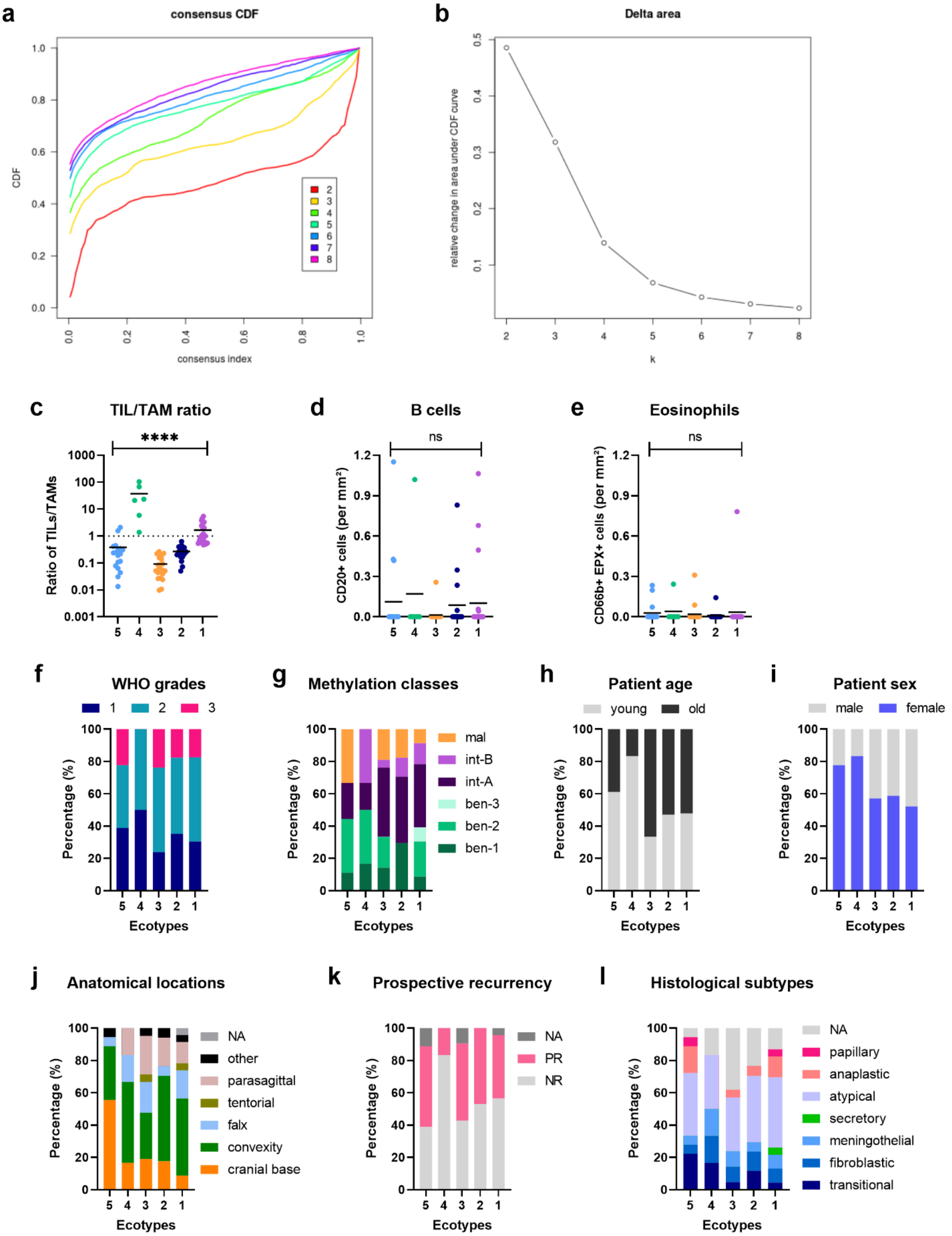
a
consensus CDF
CDF
consensus index
b
Delta area
relative change in area under CDF curve
k
c
TIL/TAM ratio
****
Ratio of TILs/TAMs
d
B cells
ns
CD20+ cells (per mm²)
e
Eosinophils
ns
CD66b+ EPX+ cells (per mm²)
f
WHO grades
g
Methylation classes
mal
int-B
int-A
ben-3
ben-2
ben-1
h
Patient age
young
old
i
Patient sex
male
female
Percentage (%)
Ecotypes
j
Anatomical locations
NA
other
parasagittal
tentorial
falx
convexity
cranial base
k
Prospective recurrency
NA
PR
NR
l
Histological subtypes
NA
papillary
anaplastic
atypical
secretory
meningothelial
fibroblastic
transitional

Suppl. Figure S6: Immune ecotype analysis in newly diagnosed meningioma.

a-b Analysis of immune ecotypes in newly diagnosed MGMs using consensus clustering showing (**a**) cumulative distribution function (CDF) plot and (**b**) delta area plot. **c-e** Log10-transformed cell densities by immune ecotype showing (**c**) ratio of TIL/TAM densities, (**d**) B cell infiltration, and (**e**) eosinophil infiltration. **f-l** Distribution of (**f**) WHO grades, (**g**) methylation (sub)classes, patient (**h**) age and (**i**) sex, (**j**) anatomical locations, (**k**) prospective recurrency state, and (**l**) histological subtypes by immune ecotype. Statistical significance was calculated ordinary one-way ANOVA tests in (c-e), and Fisher's exact test in (f-l). ben, benign; CDF, cumulative distribution function; intermediate, int; mal, malignant; MGM, meningioma; NA, not available; NR, non-recurring; PR, prospectively recurring; TAM, tumor-associated macrophage; TCC, total cell count; TIL, tumor-infiltrating T lymphocyte. Statistical significance: ****, $P<0.0001$.

Supplementary Table S1: Impact of neutrophil infiltration on progression-free survival. Multivariate survival analysis (Cox proportional hazard model).

		N	HR	95%-CI	*P*-VALUE
**NEUTROPHIL INFILTRATION**	low	45	1.00		
	high	45	2.11	1.08-4.11	**0.03***
**METHYLATION CLASS**	benign	32	1.00		
	intermediate	44	6.92	2.36-20.26	**<0.001*** **
	malignant	14	10.24	3.11-33.79	**<0.001*** **
**SEX**	female	56	1.00		
	male	34	1.77	0.93-3.38	0.08

Results of the multivariate analysis for the progression-free survival of newly diagnosed meningioma cases calculated using Cox proportional hazard model. Abbreviations: 95%-CI, lower and upper border of 95% confidence interval; HR, hazard ratio; n, number.

Supplementary Table S2: Impact of immune ecotype on progression-free survival. Multivariate survival analysis (Cox proportional hazard model).

		N	HR	95%-CI	*P*-VALUE
**IMMUNE ECOTYPE**	1	22	1.00		
	2	17	2.14	0.77-5.96	0.146
	3	17	4.71	1.57-14.18	**0.006****
	4	6	0.46	0.06-3.74	0.471
	5	21	3.09	1.18-8.11	**0.022***
**METHYLATION CLASS**	benign	32	1.00		
	intermediate	38	8.83	2.90-26.87	**<0.001*****
	malignant	13	9.40	2.78-31.76	**<0.001*****
**SEX**	female	51	1.00		
	male	32	2.23	1.04-4.76	**0.038***

Results of the multivariate analysis for the progression-free survival of newly diagnosed meningioma cases calculated using Cox proportional hazard model. Abbreviations: 95%-CI, lower and upper border of 95% confidence interval; HR, hazard ratio; n, number.
